# Risk factors and epidemiology of *Clostridium difficile* infection in hematopoietic stem cell transplant recipients during the peritransplant period

**DOI:** 10.1111/tid.12649

**Published:** 2017-02

**Authors:** Sol del Mar Aldrete, Colleen S. Kraft, Matthew J. Magee, Austin Chan, Don Hutcherson, Amelia A. Langston, Brian I. Greenwell, Eileen M. Burd, Rachel Friedman-Moraco

**Affiliations:** 1 Department of Medicine, Division of Infectious Diseases, Emory University, Atlanta, GA, USA; 2 Department of Pathology and Laboratory Medicine, Emory University, Atlanta, GA, USA; 3 Division of Epidemiology and Biostatics, Georgia State University, Atlanta, GA, USA; 4 Department of Medicine, Division of Infectious Diseases, Duke University, Durham, NC, USA; 5 Department of Pharmacy, Emory University Hospital, Winship Cancer Institute, Atlanta, GA, USA; 6 Department of Hematology and Medical Oncology, Emory University, Atlanta, GA, USA

**Keywords:** *Clostridium difficile*, hematopoietic stem cell transplant, peritransplant

## Abstract

**Background:**

Hematopoietic stem cell transplant (HSCT) recipients represent a high-risk group for developing *Clostridium difficile* (CD) infection (CDI). We aimed to identify specific risk factors for CDI in an HSCT patient population during the peritransplant period.

**Methods:**

We performed a case–control study within a cohort of HSCT patients who received a transplant from November 2010 to March 2013. Cases had a clinical presentation compatible with CDI and a positive stool sample Xpert^®^ *C. difficile* test. Controls were CDI negative and matched on age, gender, and transplant type. Peritransplant period was defined as −30 days or time of stem cell mobilization maneuver to 30 days post transplant in autologous SCT or 90 days post transplant in allogeneic SCT.

**Results:**

Of 781 HSCTs performed during the study period, 650 (83.2%) had a stool sample submitted for CD testing. Eight-six (13.2%) cases with CDI were identified. Most of the cases were diagnosed within a week after transplantation (median of 5 days). In adjusted analysis, prior hospitalization (odds ratio [OR]: 2.01, 95% confidence interval [CI] 1.2-3.36), prior cephalosporin administration (OR 2.72, 95% CI: 1.54-4.83), and prior chemotherapy (OR: 3.26, 95% CI: 1.92-5.5) were significantly associated with CDI.

**Conclusions:**

Hospitalization, and prior antibiotic and chemotherapy use are risk factors that are not easily modifiable, which emphasizes the need to start investigating preventive or prophylactic strategies in this high-risk population.

## 1 | INTRODUCTION

Toxigenic *Clostridium difficile* (CD) is now recognized as the most common cause of infectious healthcare-associated diarrhea.^1^ A recently published surveillance study estimated that CD infection (CDI) was responsible for almost half a million infections and approximately 29 000 deaths in 2011 in the United States.^2^ Clinical manifestations of CDI range from symptomless carriage to fulminant colitis.^3^ Hematopoietic stem cell transplant (HSCT) patients can be particularly susceptible to CDI owing to the presence of multiple risk factors including prolonged hospitalization, multiple antibiotic regimens including frequent use of prophylactic antibiotics and treatment of infectious complications, use of chemotherapeutic agents, and gastrointestinal tract mucosal damage secondary to conditioning regimens/radiation or gastrointestinal graft-versus-host disease (GVHD).^4,5^ One tertiary medical center found that HSCT patients had ninefold higher rates of CDI compared to the general population (24 vs 2.6 per 10 000 patient days).^6^

The number of HSCT performed in the United States continues to increase every year, with recent estimates of a total of 19 200 transplants performed in 2013.^7^ The most common indications are multiple myeloma and lymphoma, accounting for 52% of all HSCTs; acute myeloid leukemia and myelodysplasia are the most common indications for allogeneic transplants.^8^ Despite the growing numbers of HSCT performed in the United States and the high-risk nature of this population, no modifiable risk factors for CDI have been identified.

The peritransplant period appears to be an especially high-risk time for acquisition of CDI.^9^ The objective of our study was to identify patient characteristics, antibiotic regimens, and chemotherapy or conditioning regimens associated with development of CDI during the peritransplant period among HSCT patients.

## 2 | METHODS

### 2.1 | Patient population

This study was approved by the Emory University Institutional Review Board. From November 1, 2010 to March 31, 2013 a total of 781 HSCTs were performed at Emory University Hospital. All transplants were performed on the high-efficiency particulate air filtered Bone Marrow Transplant inpatient unit.

Of all patients who received a HSCT, 650 (83.2%) had a stool sample submitted for CD testing. Whenever a stool sample was submitted, patients were placed on enteric contact isolation with soap and water handwashing, until the CD test results were back. All patients received prophylactic levofloxacin (or a substitute, if allergic), acyclovir and fluconazole (autologous population), or micafungin (allotransplant population) starting 1 day before the HSCT. Levofloxacin was continued through the engraftment period or until febrile, at which point, most patients were started on standard empiric antibiotics (cefepime ± intravenous vancomycin).

### 2.2 | Study design

We performed a retrospective, case–control study within the cohort of HSCT recipients who received transplants from November 1, 2010 to March 31, 2013. Eligible patients for this study included all adults (18 years and older) who had a stool sample submitted to the laboratory for CD testing within the time indicated above. Case patients were defined as HSCT recipients with CDI in the peritransplant period indicated by symptoms or signs of CDI (any of the following: fever, leukocytosis, diarrhea, or abdominal pain)^10^ and a positive Xpert^®^ (Cepheid, Sunnyvale, CA, USA) *C. difficile* toxin B gene test during the index admission. Control patients were defined as HSCT recipients during the study period who had a stool sample submitted in the peritransplant period that was Xpert^®^ *C. difficile* negative. Where available, cases were matched 1:3 to controls by gender, age (within 7-10 years), and by transplant type (allogeneic vs autologous); eight cases were matched 1:2 because of a lack of suitable controls.

Covariates collected from study patients included clinical presentation (such as presence/absence of diarrhea, fever, abdominal pain, radiographic changes, etc.), administration of granulocyte colony-stimulating factor, presence of mucositis (grade III or higher), GVHD, antibiotics administered, conditioning regimen, and cytotoxic chemotherapy administered within 60 days prior to the index admission. The peritransplant period was considered to be from −30 days or time of stem cell mobilization maneuver to 30 days post transplant in autologous SCT or 90 days post transplant in allogeneic SCT. The conditioning regimen was defined as the preparative regimen (combination of chemotherapeutic agents with or without radiation) administered before the transplant. Transplant day 0 was the day that the cells were infused. The index admission was defined as the admission closest to the time of SCT and when the patients were tested for CDI. In most patients, this coincided with their transplant admission.

### 2.3 | Statistical analysis

Data were analyzed using SAS version 9.4 (SAS Institute, Cary, NC, USA). The χ^2^ test (for categorical variables) and Wilcoxon rank-sum test (for continuous variables) was used to assess the association between patient characteristics and antibiotic regimens with the development of CDI. Conditional logistic regression was used to estimate the adjusted association (adjusted odds ratio [ORs] and 95% confidence intervals [CIs]) between patient covariates and acquisition of CD, controlling for matched patient characteristics (age, gender, and type of transplant). A two-sided *P* <.05 was considered statistically significant for all analyses.

## 3 | RESULTS

### 3.1 | Study population

A total of 781 HSCTs were performed during the study period. Of patients with transplants, 650 (83.2%) had a stool sample submitted for CDI testing. Autologous transplants were performed in 507 and allogeneic in 143 (including 2 syngeneic and 2 cord blood). Of the 650 tested, 86 (13.2%) cases were identified who developed CDI and 564 (86.7%) who had a stool sample submitted but did not develop CDI during the study period. Our control population was sampled from this pool of 564 patients with a negative CD test. Most of the population that was tested for CDI received an autologous transplant (78%, 507/650) but a higher proportion of allogeneic patients developed CDI during the study period (17.5% [25/143] allogeneic vs 12% [61/507] autologous [*P*=.044]). We included 82 of the 86 identified cases in the analysis, as 2 allogeneic HSCT cases had no suitable control patients and the other 2 were diagnosed with CDI after the peritransplant period.

The mean age of the case patients was 53.7 years (range 22-74 years), and 50 (60.9%) were male. Multiple myeloma was the most common reason for transplantation in cases (43/82, 52.4%). Most of the cases of CDI were diagnosed within a week after transplantation (median: 5 days, interquartile range [IQR]: 3-8 days).

Besides diarrhea, other common symptoms of CDI were fever (46/82 [56.1%]) and abdominal pain (29/82 [35.3%]). Most cases had leukopenia at the time of diagnosis with a median white blood cell count of 1050 cells/mm^3^ (IQR; 300-3300 cells/mm^3^). None of the cases required surgical intervention. In regard to treatment, 80 (97.5%) received oral vancomycin either alone (53/82 [64.6%]) or in combination with metronidazole (16 received oral metronidazole and 11 intravenous metronidazole); the remaining 2 patients received oral metronidazole only. Six of the cases (7.3%) had recurrence of CDI. Fifity-one (60.7%) had complete resolution of their diarrhea, 16 (19.5%) had improvement, and 8 (9.7%) failed to resolve. No data were available on resolution of symptoms in 7 patients. Six of the cases (7.3%) had recurrence of CDI.

### 3.2 | Case–control analysis

Demographic and clinical characteristics of the cases and controls are shown in Table 1. Cases were similar to controls with respect to age, gender, and type of transplant, demonstrating that matching was performed correctly.

From the univariate analysis, CDI was more frequent in patients hospitalized in the last 60 days (53.6% vs 36.7%; *P*<.01), and having received chemotherapy 60 days before the index admission (58.5% vs 29.9%; *P*<.01). When looking at mortality within 100 days of the CD test, we observed more deaths in the cases as compared to the controls (7.3% vs 2.1%; *P*=.02). Prior antibiotic administration overall was not significantly different between cases and controls but, when analyzing by each type of antibiotic, cephalosporins were more frequently administered (36.5% in cases vs 17.7% in controls; *P*<.01) in the cases and quinolones less frequently administered (84.1% vs 92.8%; *P*=.02) (Table 2). The conditioning regimen was not significantly different between the two groups and, in the allogeneic patients, no difference was seen between myeloablative and reduced-intensity regimens (data not shown). Nonetheless, some cytotoxic chemotherapy regimens received 60 days before the index admission were more frequently seen in the patients who developed CDI (Table 3).

From the adjusted analysis, hospitalization prior to HSCT, specific chemotherapy regimens, and cephalosporin administration remained statistically significant (Table 4). Cases with CDI were more likely to have been hospitalized in the last 60 days (OR: 2.01, 95% CI: 1.2-3.36) and received cephalosporins (OR: 2.72, 95% CI: 1.54-4.83) compared to controls. With respect to chemotherapy, VDT-PACE (bortezomib, dexamethasone, thalidomide, cisplatin, doxorubicin, cyclophosphamide, and etoposide) (OR: 3.33, 95% CI: 1.35-8.20) and ICE (ifosfamide, carboplatin, and etoposide) ± rituximab (OR: 4.17, 95% CI: 1.36-12.7) regimens were significantly more common among cases. In addition, cases were more likely to have received dexamethasone-containing regimens (OR: 3.23, 95% CI: 1.7-6.15) or bortezomib-containing regimens (OR: 3.64, 95% CI: 1.84-7.18).

## 4 | DISCUSSION

Over a decade ago, the incidence of CDI in HSCT recipients was believed to be low and not a true concern in this population.^11^ However, in the last 10 years, the reported incidence has been as high as 18% in the allogeneic HSCT population^6^ and its impact on the transplant period has been shown to be significant.^12^

In our study, we found that CDI affected 13.2% of our HSCT population during the peritransplant period; 12% in autologous HSCT recipients and 17.5% in allogeneic recipients. These results are in contrast with a recent meta-analysis of CDI in HSCT recipients that reported a prevalence of 5.2% (95% CI: 3.8%-6.9%) in autologous recipients and 9.3% (95% CI: 7.0%-11.9%) in allogeneic recipients.^13^ We hypothesize that our higher incidence is related to two main factors: the use of molecular testing, and our study period, which looked at the peritransplant period only.

We confirmed what previous studies^9,14^ have noted, which is that the first week after a transplantation is the highest for risk of developing CDI, with a median of 5 days (IQR: 3-8 days) from the time of transplant to the diagnosis of CDI. This short interval might suggest that these patients are already colonized with CD and not necessarily that they acquired it in during the transplant admission.

We also found higher rates of CDI in the allogeneic HSCT recipients compared to the autologous HSCT recipients (17.5% vs 12%), which has also been found in previous studies.^6,15,16^ This difference is likely multifactorial and related to length of hospital stay, longer period of neutropenia, antibiotic exposure, and conditioning regimen. When we looked at length of hospital stay in days, the mean was significantly different between the allogeneic and autologous population (25.6 vs 17.8; *P*<.01). This difference was not observed when comparing length of hospital stay between cases and controls (20.5 vs 19.7; *P*=.46). A larger study by Shah et al.^17^, which analyzed national trends from 2001 to 2010 of the incidence and impact of CDI on hospitalization, found a difference in the mean length of stay for CDI patients for both autologous and allogeneic HSCT recipients. The fact that we were unable to detect a difference in length of stay between patients who developed CDI and those who did not was likely a result of our small sample. Another interesting point from the study by Shah et al.^17^ was the significantly higher in-hospital mortality in patients with CDI vs without CDI in autologous (4.0 vs 2.2%; *P*=.0003) and allogeneic (12.7 vs 9.2%; *P*=.0008) transplant recipients. Our study also showed a higher 100-day mortality in the cases compared to controls (7.3% vs 2.1%; *P*=.02).

Not enough data were captured to be able to calculate a comorbidity score (ie, Charlson comorbidity scoring system), so it can only be inferred that cases might represent a subgroup with higher comorbidities or more advanced disease and therefore higher mortality. Only one of the deaths in the cases was directly attributed to CDI.

In addition to confirming risk factors previously described, we found that having been hospitalized or receiving cytotoxic chemotherapy in the previous 60 days was a risk factor for developing CDI. The role of chemotherapy in the development of CDI has been better characterized in patients with solid organ tumors who receive chemotherapy.^18^ Anand and Glatt^19^ reviewed 23 cases published in the literature of chemotherapy being associated with CDI, in the absence of antibiotic therapy, and common offenders were found to be cisplatin, bleomycin, cyclophosphamide, doxorubicin, methotrexate, etc. The pathogenesis for this is not completely clear, but it is believed to be a result of alterations in the colonic microbiome or inflammatory changes related to the administration of these agents. When we grouped the chemotherapy regimens, we found an association between dexamethasone- and bortezomib-containing regimens with CDI. This association might be driven by the immunosuppressive effect on cell-mediated immunity both medications have^20^ or the fact that VDT-PACE is usually a regimen reserved for myeloma patients with advance disease and this may point toward a more susceptible population. Steroid use has also been associated with a twofold increase in mortality in CDI patients, especially when steroids were given for hematologic, endocrine, or post-transplant indications.^21^

Contrary to what has been described,^9^ we found no significant correlation between the development of GVHD and CDI. We hypothesize this might be related to the introduction of less-intensive conditioning regimens and shorter hospitalizations in the last few years.

It is interesting to note that almost all of our patients (97.6%) were started on oral vancomycin instead of metronidazole, which is the standard first-line therapy for mild-to-moderate CDI.^22^ This observation might reflect changes in practice at our institution to treat high-risk (ie, presence of neutropenia) or severe cases with oral vancomycin as first-line therapy, given recent evidence of superiority of vancomycin in patients with severe CDI.^23^

The strengths of this study are that it is a case–control study on a large cohort in a tertiary care center. Also, our study is one of the first to our knowledge to analyze CDI cases diagnosed via molecular testing. Given the current concerns for the impact, this type of diagnostic assay has on diagnosis, this study may illustrate what to expect, although further study is needed.

Our study has some limitations. First, we employed a retrospective case–control study design and we relied on adequate documentation in the electronic medical record by the primary providers. Misclassification of some patient covariates was likely. Second, as polymerase chain reaction assay was used for CDI diagnosis, some patients may have been misclassified as cases who had toxigenic CD colonization. A recent study by Jain et al.^24^ showed that the overall rates of colonization with CD at hospital admission for HSCT patients was 29.3%, of which 12% were caused by a toxigenic strain. However, it is unclear how representative their findings are, given the fact that only 30% of their total cohort participated in the study and their use of culture to isolate toxigenic CD.

Overall, despite identification in our study of risk factors such as previous hospitalization, certain chemotherapy regimens, and antibiotics, none of these are easily modifiable factors. In order to decrease the burden of CDI in this population, it will be necessary to implement prevention strategies such as stool banking pre-chemotherapy for fecal microbiota transplant^25^ or administration of fidaxomicin,^26^ which are currently being evaluated for primary prevention of CDI.

## Figures and Tables

**TABLE 1:** Demographic and clinical characteristics of cases and controls

Characteristics	Case patients with CDI (n=82)	Control patients (n=237)	*P*-value
Age in years, mean (range)	53.7 (22-74)	54.3 (21-75)	
Male gender, no (%)	50 (60.9%)	143 (60.3%)	.91

Race or ethnicity			.45

White	51 (62.2%)	166 (70%)	
Black	26 (31.7%)	58 (24.5%)	
Other	5 (6%)	13 (5.5%)	

Transplant type			.97

Autologous	59 (71.9%)	171 (72.1%)	
Allogeneic	23 (28%)	66 (27.8%)	

Underlying disease			.61

Multiple myeloma	43 (52.4%)	116 (48.9%)	
Lymphoma	14 (17%)	54 (22.7%)	
Leukemia	20 (24.4%)	58 (24.4%)	
Other^a^	5 (6.1%)	9 (3.8%)	

Mucositis^b^	21 (25.6%)	64 (27%)	.80

GVHD	4 (4.8%)	11 (4.6%)	.93

Prior antibiotics^c^	80 (97.5%)	229 (96.6%)	.67

Prior hospitalization^c^	44 (53.6%)	87 (36.7%)	**<.01**

Prior chemotherapy^c^	48 (58.5%)	71 (29.9%)	**<.01**

Bold values signify statistical significance at a *P* <.05.

a Other includes: amyloidosis, aplastic anemia, sickle cell anemia, germ cell tumor, metastatic testicular cancer, Sezary disease.

b Mucositis grade 3 or higher.

c Previous 60 days before index admission.

CDI, *C. difficile* infection; GVHD, graft-versus-host disease.

**TABLE 2:** Recent antibiotic use (univariate analysis)

Antibiotic	Cases (n=82)	Controls (n=237)	*P*-value
Penicillin	6 (7.3%)	31 (13%)	.16
Cephalosporin	30 (36.5%)	42 (17.7%)	**<.01**
Other β-lactam	7 (8.5%)	9 (3.8%)	.09
Quinolones	69 (84.1%)	220 (92.8%)	**.02**
Vancomycin IV	15 (18.2%)	29 (12.2%)	.17
Other	11 (13.4%)	50 (21.1%)	.12

Bold values signify statistical significance at a *P* <.05.

IV, intravenous.

**TABLE 3:** Prior cytotoxic chemotherapy (univariate analysis)

Chemotherapy	Cases (n=82)	Controls (n=237)	*P*-value
VDT-PACE^a^	10 (12.2%)	9 (3.8%)	**<.01**
ICE^b^ ± Rituximab	9 (11%)	8 (3.4%)	**<.01**
Hyper-CVAD^c^	5 (6.1%)	7 (2.9%)	.19
VDCEP^d^	4 (4.8%)	4 (1.7%)	.11
R-VEPTA^e^	2 (2.4%)	1 (0.4%)	.10
Decitabine	1 (1.2%)	6 (2.5%)	.48
Cyclophosphamide	3 (3.6%)	5 (2.1%)	.44
Other^f^	18 (21.9%)	31 (13%)	.05
Dexamethasone-containing regimen	22 (26.8%)	23 (9.7%)	**<.01**
Rituximab-containing regimen	8 (9.7%)	12 (5%)	.13
Bortezomib-containing regimen	24 (29.2%)	27 (11.4%)	**<.01**

Bold values signify statistical significance at a *P* <.05.

a Bortezomib, dexamethasone, thalidomide, cisplatin, doxorubicin, cyclophosphamide, and etoposide.

b Ifosfamide, carboplatin, and etoposide.

c Hyper-fractionated cyclophosphamide, vincristine, doxorubicin, and dexamethasone.

d Bortezomib, dexamethasone, cyclophosphamide, etoposide, and cisplatin.

e Rituximab, vinorelbine, paclitaxel, etoposide, cisplatin, and cytarabine.

f Thalidomide, vorinostat, cytarabine, or rituximab, etc.

**TABLE 4:** Conditional logistic regression matched odds ratio for patient characteristics

Characteristic	OR (95% CI)
Mucositis (grade 3 or higher)	0.85 (0.43-1.65)
GVHD	1.07 (0.30-3.81)
Prior hospitalization^a^	2.01 (1.2-3.36)
Prior chemotherapy^a^	3.26 (1.92-5.5)
Antibiotics	
Penicillin	0.53 (0.21-1.32)
Cephalosporin	2.72 (1.54-4.83)
Other beta-lactam	2.49 (0.8-7.69)
Quinolones	0.44 (0.21-0.94)
Vancomycin IV	1.64 (0.83-3.25)
Chemotherapy	
VDT-PACE^b^	3.33 (1.35-8.20)
ICE^c^ ± Rituximab	4.17 (1.36-12.7)
Other	1.9 (0.99-3.75)
Dexamethasone regimen	3.23 (1.7-6.15)
Bortezomib regimen	3.64 (1.84-7.18)

a Previous 60 days before index admission.

b Bortezomib, dexamethasone, thalidomide, cisplatin, doxorubicin, cyclophosphamide, and etoposide.

c Ifosfamide, carboplatin, and etoposide.

OR, odds ratio; CI, confidence interval; GVHD, graft-versus-host disease; IV, intravenous.

## References

[1] McDonald LC, Killgore GE, Thompson A, An epidemic, toxin gene-variant strain of *Clostridium difficile*. N Engl J Med. 2005;353:2433–2441. 16322603 10.1056/NEJMoa051590

[2] Lessa FC, Mu Y, Bamberg WM, Burden of *Clostridium difficile* infection in the United States. N Engl J Med. 2015;372:825–834. 25714160 10.1056/NEJMoa1408913 PMC10966662

[3] Bartlett JG. Clinical practice. Antibiotic-associated diarrhea. N Engl J Med. 2002;346:334–339. 11821511 10.1056/NEJMcp011603

[4] Bobak D, Arfons LM, Creger RJ, Lazarus HM. *Clostridium difficile*-associated disease in human stem cell transplant recipients: Coming epidemic or false alarm? Bone Marrow Transplant. 2008;42:705–713. 18836490 10.1038/bmt.2008.317

[5] Chopra T, Alangaden GJ, Chandrasekar P. *Clostridium difficile* infection in cancer patients and hematopoietic stem cell transplant recipients. Expert Rev Anti Infect Ther. 2010;8:1113–1119. 20954878 10.1586/eri.10.95

[6] Chopra T, Chandrasekar P, Salimnia H, Heilbrun LK, Smith D, Alangaden GJ. Recent epidemiology of *Clostridium difficile* infection during hematopoietic stem cell transplantation. Clin Transplant. 2011;25:E82–E87. 20973823 10.1111/j.1399-0012.2010.01331.x PMC3860287

[7] Center for International Blood and Marrow Transplant Research. Transplant Activity Report Covering 2009-2013. U.S. Department of Health and Human Services, Health Resources and Services Administration, Healthcare Systems Bureau; 2015.

[8] Pasquini MC, Zhu X. Current uses and outcomes of hematopoietic stem cell transplantation: CIBMTR Summary Slides; 2015. Available at: http://www.cibmtr.org.

[9] Willems L, Porcher R, Lafaurie M, *Clostridium difficile* infection after allogeneic hematopoietic stem cell transplantation: Incidence, risk factors, and outcome. Biol Blood Marrow Transplant. 2012;18:1295–1301. 22387347 10.1016/j.bbmt.2012.02.010

[10] Dubberke ER, Burdette SD; AST Infectious Diseases Community of Practice. *Clostridium difficile* infections in solid organ transplantation. Am J Transplant. 2013;13(Suppl 4):42–49. 23464997 10.1111/ajt.12097

[11] Tomblyn M, Gordon L, Singhal S, Rarity of toxigenic *Clostridium difficile* infections after hematopoietic stem cell transplantation: Implications for symptomatic management of diarrhea. Bone Marrow Transplant. 2002;30:517–519. 12379891 10.1038/sj.bmt.1703703

[12] Alonso CD, Marr KA. *Clostridium difficile* infection among hematopoietic stem cell transplant recipients: Beyond colitis. Curr Opin Infect Dis. 2013;26:326–331. 23806895 10.1097/QCO.0b013e3283630c4c PMC4222064

[13] Zacharioudakis IM, Ziakas PD, Mylonakis E. *Clostridium difficile* infection in the hematopoietic unit: A meta-analysis of published studies. Biol Blood Marrow Transplant. 2014;20:1650–1654. 24914822 10.1016/j.bbmt.2014.06.001

[14] Arango JI, Restrepo A, Schneider DL, Incidence of *Clostridium difficile*-associated diarrhea before and after autologous peripheral blood stem cell transplantation for lymphoma and multiple myeloma. Bone Marrow Transplant. 2006;37:517–521. 16435018 10.1038/sj.bmt.1705269

[15] Alonso CD, Treadway SB, Hanna DB, Epidemiology and outcomes of *Clostridium difficile* infections in hematopoietic stem cell transplant recipients. Clin Infect Dis. 2012;54:1053–1063. 22412059 10.1093/cid/cir1035 PMC3309884

[16] Trifilio SM, Pi J, Mehta J. Changing epidemiology of *Clostridium difficile*-associated disease during stem cell transplantation. Biol Blood Marrow Transplant. 2013;19:405–409. 23219779 10.1016/j.bbmt.2012.10.030

[17] Shah NS, Nooka AK, Kaufman JL, Evaluating risk factors and outcomes for *Clostridium difficile* infection (CDI) in stem cell transplant (SCT) recipients. Blood. 2013;122:2986.

[18] Raza S, Baig MA, Russell H, Gourdet Y, Berger BJ. *Clostridium difficile* infection following chemotherapy. Recent Pat Antiinfect Drug Discov. 2010;5:1–9. 19929843 10.2174/157489110790112608

[19] Anand A, Glatt AE. *Clostridium difficile* infection associated with anti-neoplastic chemotherapy: A review. Clin Infect Dis. 1993;17:109–113. 8353229 10.1093/clinids/17.1.109

[20] Nucci M, Anaissie E. Infections in patients with multiple myeloma in the era of high-dose therapy and novel agents. Clin Infect Dis. 2009;49:1211–1225. 19769539 10.1086/605664

[21] Das R, Feuerstadt P, Brandt LJ. Glucocorticoids are associated with increased risk of short-term mortality in hospitalized patients with *Clostridium difficile*-associated disease. Am J Gastroenterol. 2010;105:2040–2049. 20389295 10.1038/ajg.2010.142

[22] Cohen SH, Gerding DN, Johnson S, Clinical practice guidelines for *Clostridium difficile* infection in adults: 2010 update by the society for healthcare epidemiology of America (SHEA) and the infectious diseases society of America (IDSA). Infect Control Hosp Epidemiol. 2010;31:431–455. 20307191 10.1086/651706

[23] Zar FA, Bakkanagari SR, Moorthi KM, Davis MB. A comparison of vancomycin and metronidazole for the treatment of *Clostridium difficile*-associated diarrhea, stratified by disease severity. Clin Infect Dis. 2007;45:302–307. 17599306 10.1086/519265

[24] Jain T, Croswell C, Urday-Cornejo V, *Clostridium difficile* colonization in hematopoietic stem cell transplant recipients: A prospective study of the epidemiology and outcomes involving toxigenic and non-toxigenic strains. Biol Blood Marrow Transplant. 2016;22:157–163. 26211988 10.1016/j.bbmt.2015.07.020

[25] Di Bella S, Gouliouris T, Petrosillo N. Fecal microbiota transplantation (FMT) for *Clostridium difficile* infection: Focus on immunocompromised patients. J Infect Chemother. 2015;21:230–237. 25703532 10.1016/j.jiac.2015.01.011

[26] Hostler CJ, Chen LF. Fidaxomicin for treatment of *Clostridium difficile*-associated diarrhea and its potential role for prophylaxis. Expert Opin Pharmacother. 2013;14:1529–1536. 23683070 10.1517/14656566.2013.802307

